# Mesusage du tramadol par les adolescents et jeunes adultes en situation de rue

**Published:** 2012-11-18

**Authors:** Djibo Douma Maiga, Houdou Seyni, Ali Ousmane Moussa, Amadou Sidikou

**Affiliations:** 1 Faculté des Sciences de la Santé de Niamey, Niger; 2 Service de Psychiatrie. Département de médecine et spécialités médicales Hôpital national de Niamey, Niger

**Keywords:** Tramadol, Addiction, Mésusage, Adolescents, Niamey, Niger, Tramadol, Addiction, misuse, Adolescents, Niamey, Niger

## Abstract

L’objectif de cette étude transversale et descriptive était de décrire quelques caractéristiques de l’addiction au Tramadol chez les adolescents et jeunes adultes en situation de rue, rattachés aux centres d’écoute du Service Educatif, Judiciaire et préventif (SEJUP) de Niamey. Le recueil des données a porté sur les variables sociodémographiques et la consommation de tramadol. L’analyse des données a calculé les moyennes, minima, maxima et écarts types. Les résultats indiquent que : trois centres d’écoute sur huit participaient à l’étude. L’échantillon constituait 61 adolescents et jeunes adultes dont l’âge moyen était de 17,49 ans. L’analyse indique que 47 adolescents et jeunes adultes de toutes les catégories sociodémographiques (sexe, niveau d’instruction, provenance familiale ; région de provenance ; antécdents médicaux; antécédents judiciaires) étaient usagers du Tramadol. 46 étaient dépendants du Tramadol. La quantité moyenne consommée était de 1455,31 ± 901,4mg. Le mésusage du tramadol par les jeunes en situation de rue est probablement une des conséquences de la vente illicite et libre des médicaments, dont il est urgent de préciser les données médicales et sociales.

## Aux éditeurs du Journal Panafricain de Médecine

Le mésusage du Tramadol est un fait récent, les premières inquiétudes internationales ont été manifestées en 2003, par la notification de cas de trafic illicite et d’abus dans certains pays d’Afrique du Nord [1, 2]. En Afrique de l’ouest, avec le développement des pharmacies dites par terre (nom donné à la vente illicite en point fixe ou en ambulatoire de médicaments pharmaceutiques), le mésusage de certains psychotropes est noté chez les jeunes [3, 5]. Celui du Tramadol n’est pas documenté, néanmoins, il tend de nos jours, à supplanter celui des autres substances psychoactives. La présente étude, avait pour objectif de déterminer la fréquence de la consommation de Tramadol et de décrire quelques caractéristiques de l’addiction au Tramadol chez les adolescents et jeunes adultes en situation de rue, rattachés aux centres d’écoute du Service Educatif, Judiciaire et préventif (SEJUP) de Niamey.

Il s’agissait d’une étude transversale et descriptive sur l’usage du Tramadol par les adolescents et jeunes adultes en situation de rue rattachés, aux centres d’écoute du Service Educatif Judiciaire et Préventif (SEJUP) de Niamey. La population d’étude était tous les adolescents et jeunes adultes des 8 centres d’écoute que compte le SEJUP de Niamey. L’échantillon a été constitué d’adolescents et jeunes adultes immatriculés aux centres d’écoute dont les responsables répondaient favorablement pour participer à l’étude. Il s’agissait adolescents et jeunes adultes présents et volontaires lors des passages de l’enquêteur. Les données ont été recueillies, au cours d’interview directe de chaque cas avec un médecin. Elles étaient relatives à l’âge ; au sexe ; aux antécédents médicaux et chirurgicaux ; aux antécédents judiciaires ; à l’usage du Tramadol et à la consommation d’autres substances psychoactives. Tous les adolescents et jeunes adultes usagers du Tramadol ont été ensuite soumis au test de dépendance de la CIM10. L’analyse des données a été faite par SPSS (version 19). Elle a comporté le calcul des moyennes, des minima, maxima, écart types. L’étude a été approuvée par le ministère de la promotion de la femme et de la protection de l’enfant et le ministère de la justice. Le protocole a reçu, l’approbation du comité d’éthique de l’université Abdou Moumouni de Niamey. Trois centres d’écoute sur huit répondaient favorablement pour participer à l’étude. L’échantillon comportait 61 des 81 adolescents et jeunes adultes immatriculés des centres retenus.

### Caractéristiques de l’échantillon

L’âge moyen de l’échantillon était de 17,49 ans avec des extrêmes de 14 et 20 ans. Les deux sexes étaient représentés avec respectivement 70,5% pour les garcons et 29,5% pour les filles. Ils étaient également représentés dans les différents catégories sociodémographiques étudiées, avec un fort pourcentage de personnes de sexe masculin (60% et plus) sauf pour le niveau d’instruction et la région de provenance.

### Caractéristiques de la consommation et des usagers du Tramadol

L’analyse indique que 47 soit 77,04% de l’échantillon étaient usagers du Tramadol, dont 46 dépendants du Tramadol et 1 non dépendant. Toutes les catégories sociodémographiques sont représentées parmi les usagers, avec un fort pourcentage d’usagers à instruction primaire 60%, de sexe masculin et à antécédents judiciaires de gardes à vues. Le sexe féminin représentait 22,22% des usagers. Le Tramadol provenait des « pharmacies par terre ». Les comprimés utilisés étaient dosés à 50, 100, 150 et 200 mg. Le tableau 1 résume les caractéristiques de la consommation (quantité moyenne consommée, nombre de prise par jour, voie d’administration et substances associées).

**Tableau 1:** Caractéristiques de la consommation du Tramadol

Caractéristiques de la consommation		
Quantité moyenne consommée	Par jour en mg	1455,31 ± 901,4
	Mode	1200
	Minimum	300
	Maximum	4500
Nombre moyen de prise	Par jour	1,98 ± 73
**Voie d’administration**	Orale	100%
Substances associées	Café	70,5%
	Thé	9,8%
	Cigarette	67,2%
	Cannabis	77,0%
	Psychotropes (benzodiazépines, amphétamines, clomipramine)	4,9%
	Alcool et dérivés	37,7%
	Colles et solvants organiques	39,3%

La quantité moyenne de Tramadol consommée par jour était de 1455,31 mg±901,40 en deux prises (1,98 ± 737) exclusivement par voie orale

Notre étude documente la consommation de Tramadol au Niger, elle en donne la fréquence et la quantité moyenne consommée chez des adolescents et jeunes adultes en situation de rue dont le profil sociodémographique est comparable à celui des sujets décrits à risques de conduites addictives [3–5]. On notait, toutefois, une différence au niveau de l’instruction et du sexe. En effet, notre échantillon comportait une proportion plus importante d’adolescents et de jeunes d’adultes scolarisés, en raison probablement du relèvement du taux d’alphabétisation dans le pays. Il comportait également des adolescents et jeunes adultes de sexe féminin en proportion faible par rapport à celle rapportée par L’OMS [4], et l’explication pourrait en être culturelle et religieuse. L’explication de cette nouvelle addiction pourrait être la prolifération des ”pharmacies par terre”, circuit de distribution de médicaments illicites, il pourrait également s’expliquer par l’environnement social, économique, et culturel, incitateur de nouvelles conduites addictives. Une des révélations de ce travail, est la notification de conduite addictive chez les personnes de sexe féminin en situation de rue au Niger. La quantité moyenne de Tramadol consommée par jour est comparable à celle trouvée par Prakash en Irak [6]. Cette quantité moyenne est largement au dessus des doses thérapeutiques. Il est important de noter que l’ampleur du phénomène est à relativiser du fait de l’absence d’informations précises sur la prévalence des jeunes en situation de rue à Niamey. Néanmoins, on peut donner à l’étude, le crédit de mettre la lumière sur cette nouvelle addiction, et d’inciter la recherche sur les aspects quantitatifs.

## Conclusion

Le mésusage du Tramadol peut être considéré comme le symptôme d’un dysfonctionnement social. Il s’agit d’une situation naissante, dont il est urgent de préciser les données médicales et sociales.
